# Antioxidant Activities and Phytochemicals of Leaf Extracts from 10 Native *Rhododendron* Species in Taiwan

**DOI:** 10.1155/2014/283938

**Published:** 2014-06-02

**Authors:** Chi-Yang Lin, Lei-Chen Lin, Shang-Tse Ho, Yu-Tang Tung, Yen-Hsueh Tseng, Jyh-Horng Wu

**Affiliations:** ^1^Department of Forestry, National Chung Hsing University, Taichung 402, Taiwan; ^2^Department of Forestry and Natural Resources, National Chiayi University, Chiayi 600, Taiwan; ^3^Department of Life Sciences, National Chung Hsing University, Taichung 402, Taiwan

## Abstract

*Rhododendron*, one of the most famous ornamental plants in the world, is traditionally a medicinal plant. However, the potential bioactivities of native *Rhododendron* in Taiwan have not been completely studied. In this study, the results revealed that *Rhododendron pseudochrysanthum* exhibited the best antioxidant activities among 10 native *Rhododendron* species in Taiwan. Furthermore, based on a bioactivity-guided isolation principle, nine specific phytochemicals were isolated and identified as (2*R*,3*S*)-catechin (**1**), (2*R*,3*R*)-epicatechin (1′), (2*R*,3*R*)-dihydromyricetin 3-*O*-**β**-l-arabinopyranoside (**2**), (2*S*,3*S*)-taxifolin 3-*O*-**β**-l-arabinopyranoside (2′), (2*R*,3*R*)-taxifolin 3-*O*-**β**-l-arabinopyranoside (**3**), myricetin 3-*O*-**β**-d-glucopyranoside (3′), rutin (**4**), hyperoside (**5**), and quercitrin (**6**). Of these compounds, **2** and **3** were found to be major bioactive compounds, and their concentrations in the *n*-butanol (BuOH) fraction were determined to be 52.0 and 67.3 mg per gram, respectively. These results demonstrated that methanolic extracts of *Rhododendron pseudochrysanthum* leaves have excellent antioxidant activities and great potential as a source for natural health products.

## 1. Introduction

Reactive oxygen species (ROS) including superoxide radical, hydroxyl radical, singlet oxygen, and hydrogen peroxide play an important role in the initiation and progression of chronic and age-related diseases such as aging, inflammatory injury, neural disorders, diabetes, atherosclerosis, cancer, and cardiovascular disease [1]. Thus, a potential ROS scavenger may serve a role in the prevention of chronic and age-related diseases induced by ROS [2]. It has been demonstrated that the antioxidant effects of plants can be mainly attributed to phenolic compounds, such as flavonoids, phenolic acids, tannins, and phenolic diterpenes [3, 4]. Therefore, the intake of dietary antioxidants that act as ROS scavengers is expected to be effective in preventing many chronic and age-related diseases [5, 6].

The genus* Rhododendron*, which contains over 1000 species, includes flowering plants widely distributed throughout the world (with the exception of Africa and South America) [7]. Many species of the genus* Rhododendron* contain a large number of phenolic compounds and antioxidant activities that could be developed into pharmaceutical products [8]. In addition, some members of the genus are already used in traditional medicine for several ailments, especially arthritis, acute and chronic bronchitis, asthma, pain, inflammation, rheumatism, hypertension, and muscle and metabolic diseases [9–13]. In Taiwan,* Rhododendron *species are found from the lowlands to 3950 m in elevation. However, to the best of our knowledge, there is no prior report on the antioxidative phytochemicals of leaf extracts from native* Rhododendron* species in Taiwan. Thus, in this study, a number of* in vitro* assays were performed to evaluate the antioxidant activities of methanolic extracts from the leaves of 10 native* Rhododendron *species. In addition, the characteristics of bioactive phytochemicals were addressed in this study.

## 2. Materials and Methods

### 2.1. Extraction and Fractionation of 10 Native* Rhododendron* Species

**Figure 1:**
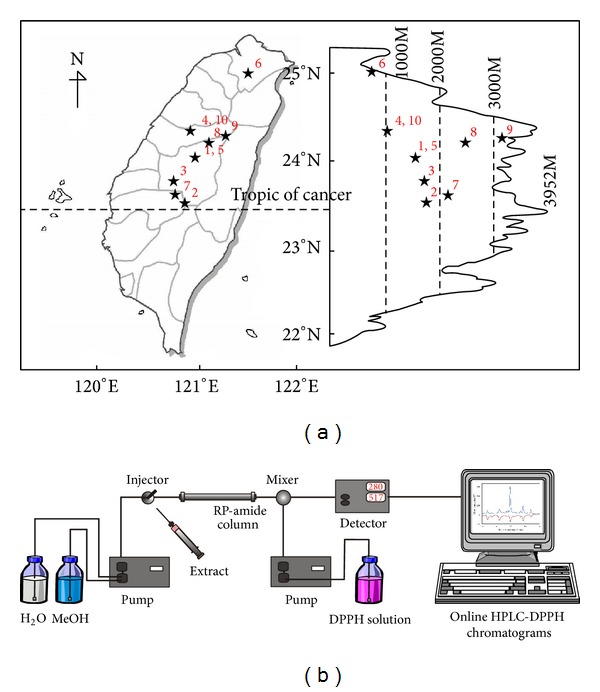
(a) The locations of 10 native* Rhododendron* species in Taiwan. (b) The instrumental setup for the HPLC-DPPH online detection of radical scavenging phytochemicals.

The leaves of 10 native* Rhododendron* species in Taiwan were collected at the end of April 2011. The collection locations of these specimens, including* R. breviperulatum* Hayata (voucher number 1),* R. ellipticum* Maxim. (voucher number 2),* R. formosanum *Hemsl. (voucher number 3),* R. kanehirai* Wilson (voucher number 4),* R. mariesii* Hemsl. and Wilson (voucher number 5),* R. oldhamii* Maxim. (voucher number 6),* R. pseudochrysanthum* Hayata (voucher number 7),* R. rubropilosum* Hayata var.* rubropilosum *(voucher number 8),* R. rubropilosum *Hayata var.* taiwanalpinum *(Ohwi) Lu, Yang and Tseng (voucher number 9), and* R. simsii *Planch. (voucher number 10), are presented in Figure 1(a). All of the voucher specimens were deposited at the herbarium of the Department of Forestry and Natural Resources, National Chiayi University (NCYU), Taiwan. The species were identified by Dr. Lei-Chen Lin (NCYU). After identification, the samples were cleaned with tap water and dried. Then, they were extracted twice with methanol by ultrasound-assisted extraction for 30 min at room temperature. The leaf extracts of 10 native* Rhododendron* species were decanted, filtered under vacuum, and either concentrated in a rotator evaporator or lyophilized. Furthermore, the resulting methanolic crude extracts of* R. pseudochrysanthum* were fractionated successively with* n*-hexane, ethyl acetate (EtOAc),* n*-butanol (BuOH), and water to yield soluble fractions of hexane, EtOAc, BuOH, and water. All the extracts were stored in an airtight container at −40°C until further use.

### 2.2. 1,1-Diphenyl-2-picrylhydrazyl (DPPH) Assay

The DPPH radical scavenging activity of the test extracts was examined according to the method reported by Ho et al. [14]. Ten microliters of each test sample in methanol, yielding a series of extract concentrations of 1, 5, 10, 50, and 100 *μ*g/mL, respectively, in each reaction, was mixed with 200 *μ*L of 0.1 mM DPPH-ethanol solution and 90 *μ*L of 50 mM Tris-HCl buffer (pH 7.4). Methanol (10 *μ*L) alone was used as the control for this experiment. After 30 min of incubation at room temperature, the reduction in DPPH radicals was measured by reading the absorbance at 517 nm using a Thermo Scientific Multiskan GO microplate reader (Vantaa, Finland). (+)-Catechin was used as the positive control. The inhibition ratio was calculated using the following equation: % inhibition = [(absorbance of control − absorbance of test sample)/absorbance of control] × 100.

### 2.3. Superoxide Radical Scavenging Assay (NBT Assay)

The measurement of superoxide radical scavenging activity was conducted according to the method of Tung et al. [15]. First, 20 *μ*L of 15 mM Na_2_EDTA in buffer (50 mM KH_2_PO_4_/KOH, pH 7.4), 50 *μ*L of 0.6 mM NBT (Nitro blue tetrazolium) in buffer, 30 *μ*L of 3 mM hypoxanthine in 50 mM KOH, 5 *μ*L of the test samples in methanol (final concentrations were 1, 5, 10, 50, and 100 *μ*g/mL, resp.), and 145 *μ*L of the buffer were mixed in 96-well microplates. The reaction was started by adding 50 *μ*L of xanthine oxidase solution in buffer (1 unit in 10 mL buffer) to the mixture. The reaction mixture was incubated at room temperature, and the absorbance at 570 nm was determined every 1 min (up to 9 min) using a microplate reader. The control was 5 *μ*L of methanol instead of the sample solution, and (+)-catechin was used as the positive control. The inhibition ratio was calculated using the following equation: % inhibition = [(rate of control reaction − rate of sample reaction)/rate of control reaction] × 100.

### 2.4. Ferrous Ion Chelating Assay

The ferrous ion chelating potential of the test samples was evaluated according to the method of Tung et al. [15]. Briefly, 200 *μ*L of the test sample in methanol (final concentrations were 125, 250, 500, 1000, and 2000 *μ*g/mL, resp.) and 740 *μ*L of methanol were added to 20 *μ*L of 2 mM FeCl_2_. The reaction was initiated by adding 40 *μ*L of 5 mM ferrozine. The mixture was shaken vigorously and permitted to rest at room temperature for 10 min. The absorbance of the solution was then measured at 562 nm. (+)-Catechin was used as the positive control. The percent inhibition of Fe^2+^-ferrozine complex formation was calculated according to the following equation: % inhibition = [(absorbance of control − absorbance of sample)/absorbance of control] × 100.

### 2.5. Reducing Power Assay

This assay was determined according to the method reported by Tung et al. [15] using (+)-catechin as the standard. Briefly, 1 mL of a reaction mixture containing 500 *μ*L of 880 *μ*g/mL test sample in 500 *μ*L of phosphate buffer (0.2 M, pH 6.6) was incubated with 500 *μ*L of potassium ferricyanide (1% w/v) at 50°C for 20 min. The reaction was terminated by adding 500 *μ*L of trichloroacetic acid (10% w/v), and the mixture was then centrifuged at 12000 g for 10 min. The supernatant solution (500 *μ*L) was mixed with distilled water (500 *μ*L) and 100 *μ*L of the ferric chloride (0.1% w/v) solution, and the optical density (OD) was then measured at 700 nm. The reducing power ability was expressed as milligrams of (+)-catechin equivalents (mg CE) per gram of sample or as micromolar of (+)-catechin equivalents (*μ*M CE) per millimolar of compound.

### 2.6. Determination of Total Phenolics

Total phenolic contents were determined according to the Folin-Ciocalteu method [15] using gallic acid as the standard. The test samples (5 mg) were dissolved in 5 mL of methanol/water (50 : 50 v/v), and the extract solution (500 *μ*L) was mixed with 500 *μ*L of 50% Folin-Ciocalteu reagent. The mixture was incubated at room temperature for 5 min, which was followed by the addition of 1.0 mL of 20% Na_2_CO_3_. After an additional 10 min of incubation at room temperature, the mixture was centrifuged for 8 min (12000 g), and the supernatant absorbance was measured at 730 nm. The total phenolic content was expressed as milligrams of gallic acid equivalents (mg GAE) per gram of sample.

### 2.7. Online HPLC-DPPH Method

The BuOH soluble fraction of* R. pseudochrysanthum* was further analyzed by the online HPLC-DPPH method. The instrumental setup is depicted in Figure 1(b). The BuOH soluble fraction (stock concentration = 20 mg/mL) was monitored by HPLC on a Jasco PU-2080 instrument (Tokyo, Japan) with a 250 × 4.6 mm i.d. and a 5 *μ*m Supelco RP-amide column (Bellefonte, PA, USA). The mobile phase consisted of solvent** A**, 100% MeOH, and solvent** B**, ultrapure water. The elution conditions were 0–5 min of 30–36%** A** and 5–50 min of 36–72%** A**, at a flow rate of 0.75 mL/min, using a Jasco MD-2010 photodiode array detector (Tokyo, Japan) at 280 nm wavelength. For the online DPPH radical scavenging analysis, the flow of DPPH reagent (50 mg/L in MeOH) was 0.75 mL/min, and the induced bleaching was detected photometrically as a negative peak at 517 nm.

### 2.8. Isolation and Identification of Bioactive Phytochemicals

Based on the bioactivity-guided isolation principle, the BuOH-soluble fraction from* R. pseudochrysanthum* had an excellent antioxidant activity. Therefore, it was separated and purified by semipreparative HPLC using a PU-2080 pump equipped with a MD-2010 multiwavelength detector and a 250 × 10.0 mm i.d. 5 *μ*m Supelco RP-amide column (Bellefonte, PA, USA). The mobile phase consisted of solvent** A**, 100% MeOH, and solvent** B**, ultrapure water. The elution conditions were 0–5 min of 30–36%** A** and 5–50 min of 36–72%** A**, at a flow rate of 2 mL/min for isolation of the BuOH fraction. The structures of the phytochemicals were identified by electrospray ionization mass (ESIMS), nuclear magnetic resonance (NMR), and circular dichroism (CD) spectrometers, and all spectral data (as shown in supplementary data; see S1–S9 in Supplementary Material available online at http://dx.doi.org/10.1155/2014/283938) were consistent with the literature [16–24]. ESIMS data were collected using a Finnigan MAT-95S mass spectrometer (San Jose, CA, USA); NMR spectra were recorded using a Bruker Avance 500 MHz FTNMR spectrometer (Rheinstetten, Germany), and CD spectra were obtained on a Jasco J-815 spectropolarimeter (Tokyo, Japan).

### 2.9. Statistical Analysis

Significant differences were calculated by Scheffe's test, and results with *P* < 0.05 were considered statistically significant. Comparisons of total phenolic contents and various antioxidant activities were carried out using Pearson's correlation test.

## 3. Results and Discussion

### 3.1. The Methanolic Extract Yields of 10 Native* Rhododendron* Species in Taiwan

The leaves of 10 native* Rhododendron* species in Taiwan yielded methanolic extracts from 6.8 to 33.4% (w/w) based on dry weight (Table 1). The yields of 7 species were higher than 15%, including* R. ellipticum* (33.4%),* R. oldhamii* (29.1%),* R. kanehirai* (22.5%),* R. breviperulatum* (18.4%),* R. formosanum* (18.2%),* R. mariesii* (18.0%), and* R. rubropilosum* var.* taiwanalpinum* (16.7%). In addition, the yields among different species showed great variability. For example, the yield of* R. ellipticum* (33.4%) was fivefold higher than that of* R. pseudochrysanthum* (6.8%).

### 3.2. Antioxidant Activities of Methanolic Extracts of 10 Native* Rhododendron* Species in Taiwan

An approach using multiple assays for screening antioxidant properties is highly advisable [25, 26]. Thus, the extracts were subjected to four different antioxidant assays employing DPPH, NBT, and ferrous ion chelating and reducing power methods. In regard to the inhibitory effects of the leaf extracts from 10 native* Rhododendron* species in Taiwan on DPPH radicals, Table 1 shows that most extracts revealed good DPPH radical scavenging activity. The concentrations required to inhibit 50% of the radical scavenging effect (IC_50_) were determined from the results of a series of tested concentrations. The IC_50_ values of crude extracts increased in the following order:* R. pseudochrysanthum* (7.5 *μ*g/mL),* R. oldhamii* (7.5 *μ*g/mL),* R. kanehirai* (7.7 *μ*g/mL),* R. breviperulatum* (8.8 *μ*g/mL),* R. rubropilosum* var.* taiwanalpinum* (10.4 *μ*g/mL),* R. formosanum* (10.7 *μ*g/mL),* R. simsii* (11.8 *μ*g/mL),* R. rubropilosum* var.* rubropilosum* (12.1 *μ*g/mL),* R. ellipticum* (14.2 *μ*g/mL), and* R. mariesii *(14.7 *μ*g/mL). In comparison with a well-known antioxidant, (+)-catechin (IC_50_ = 2.1 *μ*g/mL); the crude extracts of the* Rhododendron *species mentioned above exhibited a good DPPH radical scavenging activity. Furthermore, the crude extract of green tea showed an excellent DPPH radical scavenging activity with an IC_50_ value of 5 *μ*g/mL [5]. These results indicate that the leaf extracts of* R. pseudochrysanthum* and* R. oldhamii *would be excellent sources of natural antioxidants and merit further investigation.

Additionally, the superoxide radical scavenging activities of the leaf extracts from 10 native* Rhododendron* species in Taiwan were determined by the hypoxanthine-xanthine oxidase system. The inhibitory activities of 10 species were observed in a dose-dependent manner (Table 1), and the leaf extracts of* R. pseudochrysanthum* exhibited the highest superoxide radical scavenging activity among all of the species. The IC_50_ values of (+)-catechin,* R. breviperulatum*,* R. ellipticum*,* R. formosanum*,* R. kanehirai*,* R. mariesii*,* R. oldhamii*,* R. pseudochrysanthum*,* R. rubropilosum* var.* rubropilosum*,* R. rubropilosum* var.* taiwanalpinum*, and* R. simsii *were 16.6, 24.3, 15.7, 20.1, 33.9, 29.0, 19.6, 12.4, 24.5, 34.0, and 31.6 *μ*g/mL, respectively.

On the other hand, Table 1 shows the reducing power and ferrous ion chelating activity of the leaf extracts of the native* Rhododendron* species. The reducing powers of the different species occurred in decreasing order as follows:* R. pseudochrysanthum* (387 mg CE/g),* R. kanehirai* (328 mg CE/g),* R. oldhamii* (326 mg CE/g),* R. breviperulatum *(320 mg CE/g),* R. ellipticum *(298 mg CE/g),* R. formosanum* (282 mg CE/g),* R. simsii* (277 mg CE/g),* R. rubropilosum* var.* rubropilosum *(263 mg CE/g),* R. rubropilosum* var.* taiwanalpinum *(243 mg CE/g), and* R. mariesii* (225 mg CE/g). As for the chelating effects of the methanolic extracts on ferrous ions, the results revealed that the IC_50_ values of chelating effect for leaf extracts were in increasing order as follows:* R. kanehirai* (429.1 *μ*g/mL),* R. mariesii* (515.3 *μ*g/mL),* R. breviperulatum* (665.3 *μ*g/mL),* R. oldhamii* (714.1 *μ*g/mL),* R. rubropilosum* var.* taiwanalpinum* (774.1 *μ*g/mL),* R. rubropilosum* var.* rubropilosum* (804.9 *μ*g/mL),* R. formosanum* (928.8 *μ*g/mL),* R. simsii* (961.2 *μ*g/mL),* R. ellipticum* (1095.9 *μ*g/mL), and* R. pseudochrysanthum* (1344.4 *μ*g/mL). Comparison of the aforementioned results indicated that the ferrous ion chelating effects of the methanolic extracts did not correlate with the results from the DPPH and reducing power assays. Similar results were reported by Chua et al. [27] and Tung et al. [28]. It has been reported that an effective chelating agent can stabilize the oxidized form of the metal ion through formation of σ bonds with the metal, leading to reduce the redox potential [29]. In contrast, the free radical scavenging activities of the test samples are mainly due to their hydrogen-donating ability. Accordingly, in the present study, the discrepancy of ion chelating assay and other antioxidant assays may be due to the different mechanisms involved in different assays.

### 3.3. Total Phenolics of Methanolic Extracts of 10 Native* Rhododendron* Species in Taiwan

Table 1 shows that the contents of total phenolics in crude extracts were determined spectrometrically according to the Folin-Ciocalteu method and calculated as gallic acid equivalents (GAE). Accordingly, the total phenolic contents of the 10 native* Rhododendron* species in Taiwan were ranked in decreasing order as follows:* R. pseudochrysanthum* (319 mg GAE/g),* R. breviperulatum* (265 mg GAE/g),* R. oldhamii* (264 mg GAE/g),* R. kanehirai* (238 mg GAE/g),* R. formosanum* (222 mg GAE/g),* R. ellipticum* (221 mg GAE/g),* R. simsii* (219 mg GAE/g),* R. rubropilosum* var.* taiwanalpinum* (198 mg GAE/g),* R. rubropilosum* var.* rubropilosum* (193 mg GAE/g), and* R. mariesii* (165 mg GAE/g). This result revealed that the leaf extracts of* R. pseudochrysanthum* had the highest phenolic contents among all species of* Rhododendron*.

### 3.4. Correlation Coefficients among DPPH Radical Scavenging Activity, Superoxide Radical Scavenging Activity, Ferrous Ion Chelating Ability, Reducing Power, and Total Phenolic Contents in Extracts

Phenolic compounds had been widely evaluated for their bioactivities, especially in antioxidant activities. Thus, the correlation between the total phenolic contents and antioxidant activities has been studied in different plants [14]. In this study, correlation coefficients for total phenolic contents with the DPPH, NBT, ferrous ion chelating, and reducing power assays are shown in Table 2. These results showed that strong correlations were obtained between total phenolic contents and DPPH assay as well as total phenolic contents and reducing power, with *R* ^2^ values of 0.787 (*P* < 0.05) and 0.966 (*P* < 0.01), respectively. However, the correlations between total phenolic contents and NBT assay as well as total phenolic contents and ferrous ion chelating assay were not significant (*P* > 0.05). Accordingly, antioxidant activities of extracts, especially in DPPH radical scavenging ability and reducing power, are correlated to their phenolic contents. Furthermore, from the results obtained from the DPPH, NBT, reducing power, and total phenolic content assays, it is clear that effective antioxidants can be obtained from methanolic extracts of* R. pseudochrysanthum* leaves and proposed that phenolic compounds from the methanolic extracts may play an important role in antioxidant activities.

### 3.5. Antioxidant Activities of the Crude Extracts and Their Derived Soluble Fractions from* R. pseudochrysanthum* Leaves

**Figure 2:**
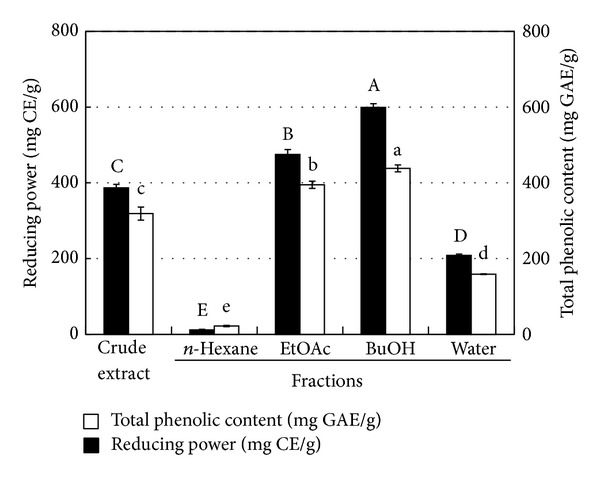
The reducing powers and total phenolic contents of methanolic extracts and their derived soluble fractions from* R. pseudochrysanthum* leaves. The results are presented as means ± SD (*n* = 3). The bars marked by different letters are significantly different at the *P* < 0.05 level according to Scheffe's test.

Based on the bioactivity-guided isolation principle, the methanolic crude extracts of* R. pseudochrysanthum* leaves were fractionated to yield soluble fractions of hexane, EtOAc, BuOH, and water. As shown in Table 3, the DPPH radical scavenging, superoxide radical scavenging, and ferrous ion chelating activities of methanolic extracts and their derived soluble fractions from* R. pseudochrysanthum* leaves increased with the increasing concentration of the test sample. The IC_50_ values of the crude extract, hexane fraction, EtOAc fraction, BuOH fraction, and water fraction of* R. pseudochrysanthum* leaves were 7.5, 71.7, 3.7, 3.3, and 15.4 *μ*g/mL for the DPPH assay; 12.4, >100, 14.2, 7.4, and 19.3 *μ*g/mL for the NBT assay; and 1344.4, >2000, 1907.8, >2000, and 281.7 *μ*g/mL for the ferrous ion chelating ability, respectively. Additionally, the reducing powers of the fractions (Figure 2) were, in decreasing order, those of the BuOH fraction (599 mg CE/g) > EtOAc fraction (475 mg CE/g) > water fraction (208 mg CE/g) > hexane fraction (12 mg CE/g). Furthermore, the total phenolic contents of the crude extract, hexane fraction, EtOAc fraction, BuOH fraction, and water fraction were 319, 22, 395, 438, and 159 mg GAE/g, respectively (Figure 2). Accordingly, except for the ferrous ion chelating effect, the antioxidant activities of* R. pseudochrysanthum* leaves can be effectively enriched in the BuOH fraction. Chelating agents are effective as secondary antioxidants because they reduce the redox potential, thereby stabilizing the oxidized forms of metal ions [30]. Therefore, the BuOH-soluble fraction from* R. pseudochrysanthum* leaves was not a good secondary antioxidant because of its poor capacity for metal ion binding, but it was an excellent primary antioxidant (or free radical scavenger). These results revealed that the BuOH-soluble fraction from the* R. pseudochrysanthum* leaves has a powerful antioxidant activity and might be a good candidate for development as a novel natural antioxidant.

### 3.6. Screening, Quantification, and Determination of the Major Antioxidants from the BuOH Fraction of* R. pseudochrysanthum* Leaves

**Figure 3:**
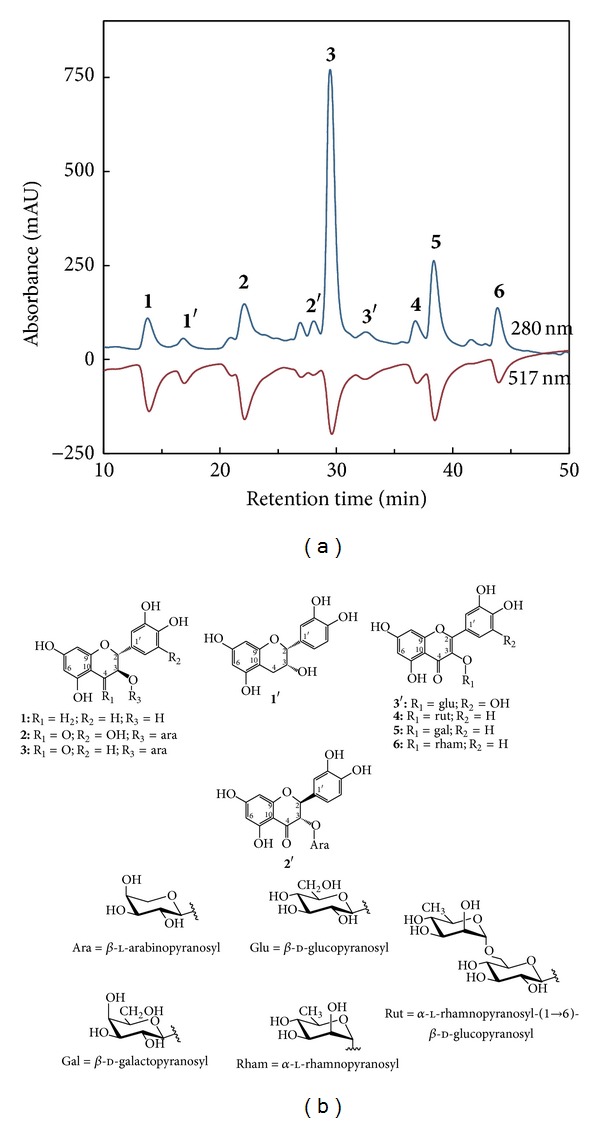
(a) The online HPLC-DPPH chromatograms of the BuOH fraction from leaves of* R. pseudochrysanthum*. (b) Isolated and identified phytochemicals: (2*R*,3*S*)-catechin (**1**), (2*R*,3*R*)-epicatechin (1′), (2*R*,3*R*)-dihydromyricetin 3-*O*-**β**-l-arabinopyranoside (**2**), (2*S*,3*S*)-taxifolin 3-*O*-**β**-l-arabinopyranoside (2′), (2*R*,3*R*)-taxifolin 3-*O*-**β**-l-arabinopyranoside (**3**), myricetin 3-*O*-**β**-d-glucopyranoside (3′), rutin (**4**), hyperoside (**5**), and quercitrin (**6**).

The online HPLC-DPPH method is a rapid assessment for detecting pure antioxidants in complex mixtures, particularly plant extracts [31, 32]. The more the absorbance decreases, the greater the potential antioxidant activity of the compound (in terms of hydrogen-donating ability) is [33].  Figure 3(a) shows combined UV (positive signals) and DPPH radical-quenching (negative signals) chromatograms under the elution conditions of the BuOH fraction from the* R. pseudochrysanthum* leaves. Nine phytochemicals, that is, (2*R*,3*S*)-catechin (**1**), (2*R*,3*R*)-epicatechin (1′), (2*R*,3*R*)-dihydromyricetin 3-*O*-**β**-l-arabinopyranoside (**2**), (2*S*,3*S*)-taxifolin 3-*O*-**β**-l-arabinopyranoside (2′), (2*R*,3*R*)-taxifolin 3-*O*-**β**-l-arabinopyranoside (**3**), myricetin 3-*O*-**β**-d-glucopyranoside (3′), rutin (**4**), hyperoside (**5**), and quercitrin (**6**), were eluted and identified (Figure 3(b)). Among them, phytochemicals** 1**–**6** showed significant hydrogen-donating capacity (negative peak), and their concentrations in the BuOH fraction were determined to be 13.2 ± 1.4, 52.0 ± 2.8, 67.3 ± 6.4, 13.4 ± 0.8, 23.8 ± 1.6, and 11.1 ± 0.9 mg per gram, respectively (Table 4). Accordingly, the results indicated that these 6 specific phytochemicals were the major antioxidants in the leaf extracts of* R. pseudochrysanthum*. The online HPLC-DPPH method can be applied for a rapid screening of antioxidants, and it is no longer necessary to isolate nonantioxidant phytochemicals.

Moreover, to determine the antioxidant activities of phytochemicals** 1**–**6**, DPPH, NBT, ferrous ion chelating, and reducing power assays were performed. As shown in Table 4, the effectiveness in DPPH radical scavenging activity was, in increasing order, that of** 4**,** 1**,** 5**,** 6**,** 3**, and** 2**, while the IC_50_ values of these compounds were 6.1, 6.8, 7.8, 9.6, 11.2, and 11.6 *μ*M, respectively. This result demonstrated that compounds** 1** and** 4** exhibited greater DPPH radical scavenging activity. In addition, the decreasing superoxide radical scavenging activity and order of phytochemicals in the NBT assay can be ranked as** 2**,** 6**,** 5**,** 1**,** 3**, and** 4**. As for the reducing power, that of compound** 3** was the greatest, followed by those of compounds** 1**,** 4**,** 5, 6**, and** 2**. Accordingly, the structure-activity relationships of flavonoid glycosides** 4**,** 5**, and** 6** were also investigated in this study. In the DPPH and reducing power assays, compound** 4**, a flavonoid with an *α*-l-rhamnopyranosyl-(1→6)-**β**-d-glucopyranosyl group in the C ring, exhibited better activities than compounds** 5** and** 6**. However, in the NBT assay, compound** 6** exhibited the best superoxide radical scavenging activity among these 3 flavonoid glycosides. According to the above results, the sugar moiety on a flavonoid plays an important role in its antioxidant activities. The effect of the sugar moiety was completely different in the DPPH and NBT assays, and this phenomenon warrants further examination in future studies.

## 4. Conclusions

In the present study, the leaf extracts of different native* Rhododendron* species in Taiwan were assayed to explore their antioxidant activities. The results indicate that a number of extracts exhibit significant antioxidant activities. Among 10* Rhododendron* species, the leaf extracts of* R. pseudochrysanthum* exhibited the strongest antioxidant activity, especially in the BuOH-soluble fraction. In total, 6 specific and excellent antioxidants were purified and identified. These results imply that the methanolic extracts of* R. pseudochrysanthum* leaves or the phytochemicals derived from these extracts could be used to prevent diseases caused by the overproduction of radicals and might also be suitable for the treatment of degenerative diseases.

## Supplementary Material

Nine phytochemicals, i.e., (*2R,3S*)-catechin (**1**), (*2R,3R*)-epicatechin (**1**'), (*2R,3R*)-dihydromyricetin 3-O-**β**-L-arabinopyranoside (**2**), (*2S,3S*)-taxifolin 3-O-**β**-L-arabinopyranoside (**2**'), (*2R,3R*)-taxifolin 3-O-**β**-L-arabinopyranoside (**3**), myricetin 3-O-**β**-D-glucopyranoside (**3**'), rutin (**4**), hyperoside (**5**), and quercitrin (**6**), were identified by electrospray ionization mass (ESIMS), nuclear magnetic resonance (NMR), and circular dichroism (CD) spectrometers, and these spectral data of the phytochemicals were shown in S1-S9, respectively.

## Figures and Tables

**Table 1:** Antioxidant activities of methanolic extracts from leaves of 10 native *Rhododendron* species.

Specimens	Yield (wt%)	IC_50_ (*μ*g/mL)			Reducing power (mg CE/g)	Total phenolic content (mg GAE/g)
		DPPH radical scavenging	Superoxide radical scavenging	Ferrous ion chelating		
*R. breviperulatum *	18.4	8.8 ± 1.6^CD^	24.3 ± 2.4^ABC^	665.3 ± 9.8^F^	320 ± 18^BC^	265 ± 2^B^
*R. ellipticum *	33.4	14.2 ± 0.9^AB^	15.7 ± 1.2^CD^	1095.9 ± 31.3^B^	298 ± 15^BCD^	221 ± 16^CD^
*R. formosanum *	18.2	10.7 ± 1.0^BCD^	20.1 ± 1.3^BCD^	928.8 ± 38.1^CD^	282 ± 5^CDE^	222 ± 16^CD^
*R. kanehirai *	22.5	7.7 ± 0.4^D^	33.9 ± 2.7^A^	429.1 ± 14.8^G^	328 ± 8^B^	238 ± 5^BC^
*R. mariesii *	18.0	14.7 ± 0.4^A^	29.0 ± 2.8^AB^	515.3 ± 4.8^G^	225 ± 11^F^	165 ± 7^E^
*R. oldhamii *	29.1	7.5 ± 0.9^D^	19.6 ± 1.0^BCD^	714.1 ± 60.0^EF^	326 ± 19^B^	264 ± 9^B^
*R. pseudochrysanthum *	6.8	7.5 ± 0.7^D^	12.4 ± 0.8^D^	1344.4 ± 18.5^A^	387 ± 9^A^	319 ± 17^A^
*R. rubropilosum* var. *rubropilosum *	11.4	12.1 ± 1.5^ABC^	24.5 ± 1.2^ABC^	804.9 ± 22.0^DE^	263 ± 9^DEF^	193 ± 7^DE^
*R. rubropilosum *var.* taiwanalpinum *	16.7	10.4 ± 1.2^BCD^	34.0 ± 1.2^A^	774.1 ± 31.9^EF^	243 ± 6^EF^	198 ± 5^DE^
*R. simsii *	9.2	11.8 ± 1.0^ABC^	31.6 ± 2.9^A^	961.2 ± 63.3^C^	277 ± 15^CDE^	219 ± 4^CD^
(+)-Catechin	—	2.1 ± 0.3^E^	16.6 ± 0.9^CD^	>2000	—	—

The results are presented as means ± SD (*n =*3). Different letters within a column indicate significant difference at the *P* < 0.05 level according to Scheffe's test. The reducing power was calculated as (+)-catechin equivalents (CE). The total phenolic content was calculated as gallic acid equivalents (GAE).

**Table 2:** Correlation coefficients among DPPH radical scavenging activity (DPPH assay), superoxide radical scavenging activity (NBT assay), ferrous ion chelating ability (Chelating assay), reducing power assay (Reducing power), and total phenolic contents (TPC) in extracts.

	DPPH assay	NBT assay	Chelating assay	Reducing power	TPC
DPPH assay	—	0.103	0.004	0.752*	0.787*
NBT assay	0.103	—	0.711*	0.577	0.581
Chelating assay	0.004	0.711*	—	0.424	0.480
Reducing power	0.752*	0.577	0.424	—	0.966**
TPC	0.787**	0.581	0.480	0.966**	—

*P < 0.05; **P < 0.01.

**Table 3:** Antioxidant activities of methanolic extracts and their derived soluble fractions from the leaves of *R. pseudochrysanthum*.

Extracts	IC_50_ (µg/mL)		
	DPPH radical scavenging	Superoxide radical scavenging	Ferrous ion chelating
Crude extract	7.5 ± 0.7^BC^	12.4 ± 0.8^C ^	1344.4 ± 18.5^B^
Hexane fraction	71.7 ± 7.4^A ^	>100	>2000
EtOAc fraction	3.7 ± 0.2^C ^	14.2 ± 1.4^BC ^	1907.8 ± 212.5^A ^
BuOH fraction	3.3 ± 0.2^C ^	7.4 ± 0.6^D^	>2000
Water fraction	15.4 ± 0.2^B ^	19.3 ± 1.5^A ^	281.7 ± 18.5^C ^
(+)-Catechin	2.1 ± 0.3^C ^	16.6 ± 0.9^AB ^	>2000

The results are presented as means ± SD (*n = *3). Different letters within a column indicate significant difference at the *P* < 0.05 level according to Scheffe's test.

**Table 4:** Antioxidant activities and contents of major phytochemicals of the BuOH fraction from leaves of *R. pseudochrysanthum*.

Phytochemicals	Contents (mg/g of BuOH fraction)	IC_50_ (*μ*M)		Reducing power (*μ*M CE/mM )
		DPPH radical scavenging	Superoxide radical scavenging	
**1**	13.2 ± 1.4	6.8 ± 0.5^CD^	45.4 ± 0.3^B^	1000 ± 34^AB^
**2**	52.0 ± 2.8	11.6 ± 0.3^A^	8.6 ± 0.3^E^	727 ± 76^D^
**3**	67.3 ± 6.4	11.2 ± 0.3^A^	45.6 ± 1.7^B^	1074 ± 35^A^
**4**	13.4 ± 0.8	6.1 ± 0.3^D^	107.5 ± 2.4^A^	917 ± 68^ABC^
**5**	23.8 ± 1.6	7.8 ± 0.7^C^	36.9 ± 0.1^C^	834 ± 14^BCD^
**6**	11.1 ± 0.9	9.6 ± 0.4^B^	24.9 ± 1.3^D^	753 ± 59^CD^

The results are presented as means ± SD (*n = *3). Different letters within a column indicate a significant difference at the *P* < 0.05 level according to Scheffe's test. The reducing power was calculated as (+)-catechin (**1**) equivalents (CE).
