# Supplementary material for: Antioxidant Activities and Phytochemicals of Leaf Extracts from 10 Native Rhododendron Species in Taiwan
Source: Evid Based Complement Alternat Med. 2014 Jun 2;2014:283938. doi: 10.1155/2014/283938 (PMC4060324; doi:10.1155/2014/283938)
Supplement: Supplementary file 1 — Nine phytochemicals, i.e., (2R,3S)-catechin (1), (2R,3R)-epicatechin (1'), (2R,3R)-dihydromyricetin 3-O-β-L-arabinopyranoside (2), (2S,3S)-taxifolin 3-O-β-L-arabinopyranoside (2'), (2R,3R)-taxifolin 3-O-β-L-arabinopyranoside (3), myricetin 3-O-β-D-glucopyranoside (3'), rutin (4), hyperoside (5), and quercitrin (6), were identified by electrospray ionization mass (ESIMS), nuclear magnetic resonance (NMR), and circular dichroism (CD) spectrometers, and these spectral data of the phytochemicals were shown in S1-S9, respectively. [file 283938.f1.pdf]

Supplementary Data (S1–S9)

S1. ^{1}H, ^{13}C, CD, and ESIMS data of (2*R*,3*S*)-catechin (**1**)

Position	δ_H[a] (multiplicity, *J* = Hz)	δ_C[a]	CD[b]	ESIMS *m/z*	Reference
2	4.57 (*d*, 7.4)	82.8	$[\theta]_{248}$ = 1335,	290.1	[16–18]
3	3.98 (*ddd*, 5.6, 7.4, 8.2)	68.8	$[\theta]_{278}$ = -9209		
4	2.84 (*dd*, 5.6, 16.1)	28.5			
	2.51 (*dd*, 8.2, 16.1)				
5	–	156.9			
6	5.94 (*d*, 1.6)	96.3			
7	–	157.6			
8	5.86 (*d*, 1.6)	95.5			
9	–	157.8			
10	–	100.9			
1’	–	132.2			
2’	6.84 (*s*)	115.3			
3’	–	146.2			
4’	–	146.2			
5’	6.72 (*d*, 8.0)	116.1			
6’	6.77 (*d*, 8.0)	120.5			

[a]Measured in CD_3OD at 400 MHz. [b]Measured in CH_3OH.
